# Characterization of Cannabis Products Purchased for Medical Use in New York State

**DOI:** 10.1001/jamanetworkopen.2022.27735

**Published:** 2022-08-19

**Authors:** Alexandra F. Kritikos, Rosalie Liccardo Pacula

**Affiliations:** 1 Heller School for Social Policy and Management, Brandeis University, Waltham, Massachusetts; 2 Leonard D. Schaeffer Center for Health Policy & Economics, University of Southern California, Los Angeles; 3 USC Institute for Addiction Sciences, University of Southern California, Los Angeles; 4 Sol Price School of Public Policy, University of Southern California, Los Angeles; 5 National Bureau of Economic Research, Cambridge, Massachusetts

## Abstract

This cross-sectional study examines the purchase of medical cannabis products made in New York State from dispensaries operated by a single integrated manufacturer and licensed retail company within the state.

## Introduction

Although the number of registered medical cannabis patients has grown,^1^ evidence regarding effective medical cannabis products and appropriate dosing by condition remains elusive.^2,3^ What little is known comes largely from self-reports,^4,5^ which presumes patient knowledge of products and specific cannabinoids, particularly delta-9 tetrahydrocannabinol (THC) and cannabidiol (CBD). In this study, we examined cannabis purchases made by registered patients in a large medical cannabis state in the US and provide new evidence regarding cannabis products.

## Methods

This cross-sectional study examined purchases of cannabis products by registered patients that were made in New York State from dispensaries operated by a single integrated manufacturer and retail company licensed within the state. New York requires dispensaries to report all patient purchases to the Department of Health with patient ID, qualifying conditions, age, and sex. These details, which are retained in the company’s data system, were provided under a Data Use Agreement to Brandeis University for the period January 2, 2016, through August 15, 2019. Institutional review board approval and informed consent were waived by Brandeis University’s Human Subjects Committee owing to deidentified data.

During this period, 27 349 patients purchased 79 885 products. To reduce noise from infrequent customers, we restricted our sample to 16 727 patients who made 2 or more visits (n = 16 727) and assessed the comparability of our sample with all registered patients in New York (Table 1). We then examined purchases by product type, cannabinoid formulation ratios, and labeled THC and CBD doses overall and by patient characteristics. Products included vaporizers, tinctures, tablets, and “other” (ie, lotions and suppositories); cannabis flower was excluded because its sale was prohibited in New York during the study period. Descriptive statistics were calculated using Stata version 16.0 (StataCorp LLC). This study followed the STROBE reporting guideline.

**Table 1.** Patient Characteristics^a^

Characteristic	Excluded sample (n = 10 498)^b^	Analytic sample (n = 16 727)^c^	Registered patients in New York State (n = 98 101)^d^
Age, y			
≤17	95 (0.9)	213 (1.3)	1138 (1.2)
18-30	1472 (14.0)	1348 (8.1)	9159 (9.3)
31-50	3761 (35.8)	4922 (29.4)	32 452 (33.1)
51-60	2140 (20.4)	3867 (23.1)	22 621 (23.1)
61-70	1710 (16.3)	3592 (21.5)	18 843 (19.2)
≥71	1320 (12.6)	2784 (16.6)	13 887 (14.2)
Male	5358 (51.0)	7916 (47.3)	NR
Female	5140 (49.0)	8811 (52.7)	NR
Registered conditions and symptoms^e^			
Amyotrophic lateral sclerosis	0	42 (0.2)	243 (0.2)
Cachexia	2 (0)	914 (5.5)	2720 (2.8)
Cancer	194 (1.8)	2196 (13.1)	11 952 (12.2)
Chronic pain	35 (0.3)	8687 (51.9)	52 118 (53.1)
Epilepsy	1 (0)	452 (2.7)	2605 (2.7)
HIV/AIDS	26 (0.2)	194 (1.2)	1085 (1.1)
Huntington disease	0	5 (0)	22 (0)
IBD	15 (0.1)	821 (4.9)	3657 (3.7)
Multiple sclerosis	2 (0)	605 (3.6)	4174 (4.3)
Neuropathy	1525 (14.5)	3688 (22.1)	14 312 (14.6)
Opioid reduction	135 (1.3)	145 (0.9)	NR^f^
Parkinson disease	3 (0)	362 (2.2)	1697 (1.7)
PTSD	385 (3.7)	752 (4.5)	2738 (2.8)
Seizures	132 (1.3)	508 (3.0)	2975 (3.0)
Severe nausea	408 (3.9)	1368 (8.2)	5293 (5.4)
Severe pain	5642 (53.7)	13 714 (82.0)	71 547 (72.9)
Severe muscle spasms	1109 (10.6)	3574 (21.4)	12 482 (12.7)
Spinal cord injury	245 (2.3)	688 (4.1)	3498 (3.6)

Abbreviations: IBD, inflammatory bowel disease; NR, not reported; PTSD, posttraumatic stress disorder.

^a^ Data are presented as No. (%) of patients.

^b^ Unweighted sample of patients who made a single purchase of a medical cannabis product. An additional 124 patients were excluded owing to incomplete data.

^c^ Unweighted sample of patients who made 2 or more purchases of a medical cannabis product.

^d^ This sample reflects data reported in the Medical Use of Marijuana Under the Compassionate Care Act Two Year Report.

^e^ Conditions are not mutually exclusive; therefore, percentages may total more than 100%.

^f^ Opioid replacement was not an approved qualifying condition for medical cannabis use during the study period.

## Results

Of the 16 727 patients in our sample, 52.6% were female and most (61.2%) were older than 50 years. The top 3 conditions or symptoms were severe pain (82.0%), chronic pain (51.9%), and neuropathy (22.1%), which is consistent with registered patients in New York overall (Table 1). Single-purchase patients were younger (18-30 years) and were less likely to have cancer and chronic pain.

Vaporizers were the most common product purchased (39.5%), as were products with a ratio of high THC to low CBD (52.4%) (Table 2). High-THC vaporizers, the most commonly purchased product overall, provide 2 mg THC per dose, whereas high-THC tinctures and tablets offer 10 mg THC per dose; the CBD dose in both products is 0.5 mg.

**Table 2.** Patient Purchases by Product Type, Ratio of Cannabinoids in Products Purchased, and Labeled Dose of THC and CBD for the Most Commonly Purchased Product^a^

Characteristic	Total	Type of product purchased				Ratio of cannabinoids in products purchased			
		Vaporizer	Tincture	Tablet	Other^b^	Equal THC:CBD	High THC:low CBD	Low THC:high CBD	Most commonly purchased product (labeled dose, mg THC/mg CBD)
Total	16 727	6603 (39.5)	6374 (38.1)	3660 (21.9)	89 (0.5)	4421 (26.4)	8759 (52.4)	3546 (21.2)	High-THC vaporizer (2.0/0.1)
Sex									
Female	8811 (52.7)	2846 (32.3)	3674 (41.7)	2229 (25.3)	62 (0.7)	2431 (27.6)	4125 (46.8)	2255 (25.6)	High-THC tincture (10.0/0.5)
Male	7916 (47.3)	3413 (43.1)	2659 (33.6)	1806 (22.8)	38 (0.5)	1757 (22.2)	4658 (58.8)	1511 (19.1)	High-THC vaporizer (2.0/0.1)
Age, y									
<21	523 (3.1)	134 (25.6)	220 (42.1)	167 (31.9)	2 (0.4)	144 (27.5)	185 (35.4)	194 (37.1)	High-CBD tincture (0.6/12.6)
21-30	1185 (7.1)	593 (50.0)	329 (27.8)	259 (21.9)	4 (0.3)	304 (25.6)	620 (52.3)	261 (22.0)	High-THC vaporizer (2.0/0.1)
31-50	4722 (28.2)	2238 (47.4)	1497 (31.7)	963 (20.4)	23 (0.5)	1152 (24.4)	2649 (56.1)	920 (19.5)	High-THC vaporizer (2.0/0.1)
51-65	5421 (32.4)	2143 (39.5)	2119 (39.1)	1128 (20.8)	32 (0.6)	1257 (23.2)	3079 (56.8)	1085 (20.0)	High-THC vaporizer (2.0/0.1)
>65	4876 (29.2)	1147 (23.5)	2189 (44.9)	1501 (30.8)	39 (0.8)	1336 (27.4)	2219 (45.5)	1321 (27.1)	High-THC tincture (10.0/0.5)
Qualifying conditions									
Cancer	2196 (13.1)	616 (28.1)	1198 (54.6)	373 (17.0)	9 (0.4)	599 (27.3)	1214 (55.3)	384 (17.5)	High-THC tincture (10.0/0.5)
HIV/AIDS	194 (1.2)	113 (58.2)	53 (27.3)	26 (13.4)	2 (1.0)	42 (21.6)	129 (66.5)	23 (11.9)	High-THC vaporizer (2.0/0.1)
ALS	42 (0.2)	7 (16.7)	22 (52.4)	13 (31.0)	0	17 (40.5)	16 (38.1)	9 (21.4)	Equal THC:CBD tincture (5.0/5.0)
Parkinson disease	362 (2.2)	64 (17.7)	187 (51.7)	111 (30.7)	0	144 (39.8)	101 (27.9)	117 (32.3)	Equal THC:CBD tincture (5.0/5.0)
Multiple sclerosis	605 (3.6)	285 (47.1)	215 (35.5)	103 (17.0)	2 (0.3)	196 (32.4)	261 (43.1)	148 (24.5)	High-THC vaporizer (2.0/0.1)
Spinal cord injury	688 (4.1)	281 (40.8)	301 (43.8)	105 (15.3)	1 (0.1)	191 (27.8)	350 (50.9)	147 (21.4)	High-THC tincture (10.0/0.5)
Epilepsy	452 (2.7)	115 (25.4)	180 (39.8)	157 (34.7)	0	82 (18.1)	115 (25.4)	255 (56.4)	High-CBD tincture (0.6/12.6)
IBD	821 (4.9)	349 (42.5)	332 (40.4)	136 (16.6)	3 (0.4)	229 (27.9)	345 (42.0)	246 (30.0)	High-THC vaporizer (2.0/0.1)
Neuropathy	3688 (22.1)	1453 (39.4)	1453 (39.4)	767 (20.8)	14 (0.4)	1041 (28.2)	1769 (48.0)	878 (23.8)	High-THC vaporizer/tincture (2.0-10.0/0.1-0.5)
Huntington disease	5 (0)	2 (40.0)	2 (40.0)	1 (20.0)	0	3 (60.0)	1 (20.0)	1 (20.0)	Equal THC:CBD vaporizer/tincture (1.0-5.0/1.0-5.0)
PTSD	752 (4.5)	405 (53.9)	234 (31.1)	109 (14.5)	4 (0.5)	196 (26.1)	439 (58.4)	117 (15. 6)	High-THC vaporizer (2.0/0.1)
Chronic pain	8687 (51.9)	3604 (41.5)	2865 (33.0)	2156 (24.8)	62 (0.7)	2181 (25.1)	4882 (56.2)	1624 (18.7)	High-THC vaporizer (2.0/0.1)
Opioid replacement	145 (0.9)	67 (46.2)	48 (33.1)	28 (19.3)	2 (1.4)	39 (26.9)	80 (55.2)	26 (17.9)	High-THC vaporizer (2.0/0.1)
Associated symptoms									
Cachexia	914 (5.5)	270 (29.5)	484 (53.0)	156 (17.1)	4 (0.4)	241 (26.4)	539 (58.9)	134 (14.7)	High-THC tincture (10.0/0.5)
Muscle spasms	3574 (21.4)	1392 (39.0)	1448 (40.5)	1448 (40.5)	20 (0.6)	1070 (29.9)	1734 (48.5)	770 (21.5)	High-THC tincture (10.0/0.5)
Severe pain	13 714 (82.0)	5485 (40.0)	5118 (37.3)	3038 (22.2)	72 (0.5)	3615 (26.4)	7305 (53.3)	2793 (20.4)	High-THC vaporizer (2.0/0.1)
Seizures	508 (3.0)	129 (25.4)	207 (40.8)	172 (33.9)	0	93 (18.3)	135 (26.6)	280 (55.1)	High-CBD tincture (0.6/12.6)
Severe nausea	1368 (8.2)	509 (37.2)	646 (47.2)	211 (15.4)	2 (0.2)	342 (25.0)	800 (58.5)	226 (16.5)	High-THC tincture (10.0/0.5)

Abbreviations: ALS, amyotrophic lateral sclerosis; CBD, cannabidiol; IBD, inflammatory bowel disease; PTSD, posttraumatic stress disorder; THC, delta-9-tetrahydrocannabinol.

^a^ Data are presented as No. (%) of patients unless indicated otherwise.

^b^ Lotions and suppositories.

Men were more likely to purchase vaporizers (43.1%), whereas women more frequently purchased tinctures (41.7%) (Table 2). High-THC products were purchased predominantly by both men and women. Patients older than 65 years and less than 21 years were more likely to purchase tinctures (44.9% and 42.0%, respectively) (Table 2). Patients in other age groups generally purchased vaporizers. Patients in all age groups except those less than 21 years preferred high-THC products, particularly patients with cancer (55.3%), HIV/AIDS (66.5%), spinal cord injury (50.9%), neuropathy (48.0%), posttraumatic stress disorder (58.4%), chronic pain (56.2%), and opioid replacement (55.2%) (Table 2). Patients with seizures and epilepsy were more likely to purchase high-CBD products (55.1% and 56.4%, respectively) (Table 2).

## Discussion

Our findings are consistent with those from self-reports^4,5^ but reveal some new insights. First, there was considerable variability in preferred product form—and hence cannabinoid mix—across most conditions. Second, the THC dose of high-THC products varied considerably (2 mg vs 10 mg). Third, although patients with epilepsy and seizures purchase high-CBD products in this market, these products also contain THC—unlike Epidiolex, the only US Food and Drug Administration–approved cannabinoid for seizures.^6^ Clinicians should understand how doses vary by product when discussing cannabis use with patients.

This study has some limitations. Our analysis only considered purchases from a single company in 1 US state and did not include cannabis flower. Purchases in other US states or from other sources could differ. Nonetheless, these data may provide a useful starting point for clinicians when discussing medical cannabis use with their patients.
